# Exploration of attitudes towards research: Operating department practitioners and theatre nurses

**DOI:** 10.1177/17504589241301204

**Published:** 2024-12-19

**Authors:** Nigel Conway, Amy Bradburn, Sarah Howcutt

**Affiliations:** 1 School of Psychology, Social Work and Public Health, Faculty of Health and Life Sciences, Oxford Brookes University, Oxford, UK; 2 Faculty of Health and Life Sciences, Northumbria University, Newcastle upon Tyne, UK; 3 School of Psychology, Social Work and Public Health, Faculty of Health and Life Sciences, Oxford Brookes University, Oxford, UK

**Keywords:** Attitudes, Attitudes towards research, Theatre Nurses’ attitudes to research, Operating Department Practitioners’ attitudes to research

## Abstract

**Background:**

A lack of awareness of who should conduct research, conflicting workload priorities, lack of research skills, lack of confidence and lack of supportive relationships are often cited as barriers for undertaking research within the perioperative environment. Building a robust research capacity for Operating Department Practitioners and Theatre Nurses to engage with and lead primary research is critical to develop perioperative clinical outcomes, and for professional and research excellence.

**Aims:**

This study aimed to explore the attitudes of Operating Department Practitioners and Theatre Nurses towards research.

**Methods:**

An online questionnaire was distributed nationally between 1 October 2022 and 31 December 2022. The questionnaire received 164 responses from 114 Operating Department Practitioners, 44 Theatre Nurses and 6 respondents identifying as other.

**Findings:**

These revealed that those with a positive attitude towards research were most likely to have obtained a Master’s level qualification (i.e. MSc) and had gained experience in research post-registration. When explored further, the overall positive attitude to research was found to be possibly linked to a belief in the usefulness of research and to prior exposure. The findings of this study can be used to help support, inform and strengthen research in clinical practice and research career aspirations.

**Conclusion:**

Analysis of the data suggests that participants holding a Master of Science degree and having previous experience of research reported a more positive attitude to research. When the specific attitudes to research were explored individually, there was also some evidence that the overall positive attitude to research was more likely to be related to a belief in the usefulness of research.

## Background

Advances in surgical technology over the last 20 years have the potential to impact on health within the United Kingdom. Topol Review (2019) identifies the need to better prepare the healthcare workforce for new approaches to meet these future advances in areas, such as healthcare economics, genomics, digital and technological developments. The Royal College of Surgeons of England (2018) also expressed concern in relation to the National Health Service (NHS) workforce needing to be prepared for a future where surgical delivery will be radically different from that of the past with the increasing introduction of new technologies. Research and leadership will be core to achieving such advances and challenges. HEE (2022) further support the strengthening of research and innovation for Operating Department Practitioners (ODPs) as part of their Allied Health Professions strategic aims. This approach to engagement in, and with, research is emphasised as a critical aspect of transforming and modernising the NHS workforce and service (National Health Service (NHS) 2024).

However, there has been no significant increase in research capacity and future leadership training for the various professional surgical groups. An example of this is the flexible multidisciplinary operating theatre workforce that will be needed to shape, perform, deliver and implement the products of research to perioperative practice. To explore and identify possible areas for development specific to these issues, the National Institute for Health Care Research (NIHR) (2020) established a number of targeted ‘Incubators’ designed to develop strategies for future-ready, research-literate leaders and workforce for the NHS. The ‘Advanced Surgical Technology Incubator’ was one such professional group made up of Allied Health Professionals (AHP), Engineering, Nursing, Paramedical and industrial research leaders. A core focus of this group was on the research aspirations, training and development of these professionals. An initial stage towards achieving this vision was to set up two subgroups; one specific to operating theatres and the other for the broader nursing profession, to gain a better understanding of current facilitators, barriers and attitudes towards research. The authors, as part of this operating theatre subgroup, focused their work on capturing the attitudes of ODPs and Theatre Nurses (TNs) towards research, to develop a greater understanding of the current situation and aspirations of both professions towards research.

Historically, the nursing profession has an established framework for research and evidence-based practice (Row 2008). Anecdotally, both ODPs and TNs appear to be engaging with research and are undertaking clinical research roles. However, this seems variable and the exact number of ODPs and TNs engaging in research or in research roles is unknown. The current undergraduate honours degree curriculum introduces both TNs and ODPs to core concepts of research, offering an opportunity to develop some knowledge, understanding and skills to build on. As the deliverers of perioperative care, ODPs and TNs are well placed to lead on research within this environment and specialist area to inform best practice. Initial literature searches as to their engagement with research indicated that they primarily focus on evidence-informed, secondary evidence-based literature approaches rather than primary research activities (Chadwick 2012, Kumah et al 2022, Moule et al 2016, Rene 2007).

Building a robust research capacity for ODPs and TNs to engage with and lead primary research is critical to develop the improvement of clinical outcomes, and for professional and research excellence (Avery et al 2020, Aveyard 2023, NHS 2023). Barriers such as poor awareness of who should do research, competing priorities from high clinical workloads, lack of research skills and confidence and lack of supportive research relationships are cited (Britton et al 2024, Whitehouse et al 2022, Williams et al 2020).

Both the Health and Care Professions Council and the Nursing and Midwifery Council are expected to take an active role in relation to research and evidence-based practice (Health and Care Professions Council (HCPC) 2022, 2023, Nursing and Midwifery Council (NMC) 2018a).

The latest HCPC Standards of Proficiency for ODPs stipulate that ODPs need to recognise a range of research methodologies relevant to their role, while understanding the research process (HCPC 2023). ODPs are also required to use research when problem-solving and to help in their decision-making and recognise the value of research when critically evaluating clinical practice. The HCPC also highlights that when appropriate, and their registrants should also involve service users within the research.

In comparison, the NMC requires their registrants to abide to the Code of Professional standards of practice and behaviour for nurses, midwives and nursing associates and Future Nurses: Standards of Proficiency for Registered Nurses (NMC 2018a, 2018b). Nurses are expected to correctly collate, handle and store research data and findings. In addition, it requires nurses to demonstrate an understanding of research methods, ethics and governance alongside critical and analytical skills to safely use, share and apply research to promote best practice (NMC 2018b).

These professional standards reflect broader key objectives to encourage more AHPs and nursing professionals’ involvement in research and also greater encouragement to engage service users in research (Health Education England, 2022, National Institute for Health Care Research 2023a). In addition, the UK Policy Framework for Health and Social Care Research emphasises the importance of research for health and social care employers identifying the importance of research in the context of improving treatments, care and other services, and their overall outcomes (Health Research Authority 2023). The policy goes further in promoting the role of such employers in creating and supporting opportunities for professional staff to take part in health and social care research. Improving ODPs and TNs engagement with research is echoed by the Department of Health and Social Care (DHSC) (2022) and by other health groups (Angus et al 2022, Comer et al 2022).

An initial scoping review was conducted and little research was found specific to ODP and TN, the results identified evidence indicative of the medical profession, non-theatre nursing and healthcare professions, such as physiotherapist attitudes to research. In addition, much of the research literature found on ‘attitudes to research’ was focused on students’ attitudes (Comer et al 2022, Kumah et al 2022). The rationale for this study was, therefore, informed by a lack of evidence specific to ODPs and TNs in relation to their engagement with research. This study focused on understanding the current attitudes of these two professional groups towards research, to inform and strengthen research in perioperative practice and support research career aspirations.

In preparation for the survey, the following sociodemographic questions (Q) were designed and used as demonstrated in the following tables. Participant responses were to be made using either a seven linear matrix, sliding scale or tick box approach.

**Overview of the survey questions:**

Table 1: Gender

**Table:** 

Q: What gender would you associate with yourself?
Options: Woman, Man, Non-binary / third gender /option to self-describe, prefer not to say.

**Table 2: Location**

**Table:** 

Q: In which part of the country do you currently work?
Options given covered all regions of Northern and Southern Ireland, Scotland, England and Wales. Participants could also select none of the these and specify.

**Table 3: Professional Group**

**Table:** 

Q: What is your professional background?
Options: ODP, Nurse, Other, please specify.

**Table 4: Level of Qualification specific to registerable qualification?**

**Table:** 

Q: What is your education and training background in relation to your current registerable qualification?
A full breadth of options was given covering all of the past and current preregistration qualifications for ODPs and nurses. Participants could also select none of these and specify.

**Table 5: Highest Level of education / qualification**

**Table:** 

Q: What is the highest education / professional award you currently hold?
A full breadth of options and levels was given covering vocational, apprenticeship and academic levels for ODPs and nurses. Participants could also select none of these and specify.

**Table 6: Level of qualification, post-qualification or postgraduate**

**Table:** 

Q: Are you currently studying towards any post-qualification or postgraduate qualification related to healthcare / medicine?
Options given covered a range of academic awards at degree, masters and doctorate levels. Participants could also select none of these and specify.

**Table 7: Experience of research**

**Table:** 

Q: Experience of research / have you undertaken research while in clinical practice post-qualification?
Participant answers to be split into the following subscales: Attitudes to research, Research Usefulness, Research Anxiety, Positive Attitudes to Research, Relevance of Research to Life, Difficulty of Research.

**Table 8: Age**

**Table:** 

Q: What age are you?	Sliding scale to be used.

## Methods

### Participants

The intended sample for this study was ODPs and TNs practising in the United Kingdom and territories. Freedom of information requests in December 2022 were sent to the HCPC and NMC, which revealed that there are 15,179 HCPC-registered ODPs, with incomplete data for the number of TNs recorded.

Discussion with professional body representatives involved with the Advanced Surgical Technology Incubator and early development of the survey (i.e. College of Operating Department Practitioners (ODPs), Association for Perioperative Practice) suggested that a sample size of 100–200 would be achievable and informed by their previous experiences of conducting surveys within their own organisations. Participants were recruited via online poster / survey information distributed through the College of Operating Department Practitioners and the Association for Perioperative Practice. Distribution via the Faculty of Perioperative Care within The Royal College of Surgeons of Edinburgh and The Royal College of Surgeons of England was also used as both organisations expressed an interest in this research project and identified that some of their members could have an ODP or TN background.

Participants were eligible if they were a qualified ODP or TN working in the United Kingdom or territories and were aged above 18 years of age.

### Data collection

Datum was collected via an online questionnaire which was distributed via an anonymous link using the Qualtrics platform (Qualtrics 2005). Data collection took place between 1 October 2022 and 31 December 2022, and organisations supporting recruitment sent at least one email reminder to eligible professionals. The analysis was conducted in IBM Social Sciences in the Social Sciences (International Business Machines IBM 2021 SPSS version 28).

Documents relating to analysis were stored on a Google Drive as per Oxford Brookes University data management protocols. Ethical approval was given by Oxford Brookes University, University Research Ethics Committee (UREC) (Registration No: L22281 / 5 September 2022). Consent from each individual participant was recorded within the questionnaire including for publication.

### Measures

The following sociodemographic attributes were collected from the participant responses to ascertain the repetitiveness of their responses to identify and collect emergent factors that were thought to be associated with attitudes to research (Table 1).

**Table 1:** Sociodemographic attributes

Categories
Gender
Location
Professional group
Level of qualification
Experience of research
Age (mean and standard deviation)

Attitudes to research were measured using the ‘Attitudes Towards Research’ (ATR) scale by Papanastasiou (2005) This scale has been designed to provide a global measure of attitudes to research with higher reliability (*r* = 0.948). The scale is also divided into subscales: research usefulness (α = 0.919), research anxiety (α = 0.918), positive attitudes to research (α = 0.929), relevance to life (α = 0.767) and research difficulty (α = 0.717).

### Treatment of data

Data were cleaned to remove incomplete or unusable responses. Item non-response was managed using imputation from the mean where two or fewer responses only were missing. Ineligible participants who did not meet the inclusion criteria were removed from the data collection, as were participants who had started the survey but who had dropped out before the final question or whose responses missed more than two arbitrary items.

Survey questions were assigned to categories created to score participant responses and aid analysis. The categories were informed by social and educational elements thought to have an impact on academic confidence (Sander & Sanders 2006) which could be applied to ODP and TNs to better understand their attitudes towards research. These categorical variables were recoded to create binary categories for the inferential analysis. Education was divided into holding a BSc versus an MSc or higher because it was hypothesised that the level of education could be a variable worth analysing.

Six enter method multiple linear regression analyses were conducted with both the global ‘Average True Range’ (ATR) measure and the ATR subscales as the dependent variables. The predictor (explanatory) variables in all models were as follows:

- Age in years
- Highest qualification – lower than MSc and MScs and higher
- Experience of research
- Gender

An a-priori sample size was calculated for multiple regression with four predictors, using the calculator provided at danielsoper.com (version 4) (Soper, 2023). Since there was little prior research with the same population, a moderate effect size was assumed (0.15) with a desired statistical power of 0.8 and an alpha level of 0.05. The minimum required sample size was 84.

## Results

In total, 218 participants agreed for their responses to be included as part of the anonymised survey. Of these, 54 were excluded either due to not fully meeting the inclusion criteria or giving incomplete information. Consequently, 164 participants were included in the survey datum analysis after the removal of contaminated data.

## Characteristics of the sample

Of the 164 final total participants (Table 2), the mean age was 42 years. However, 37 of participants were male, 112 were female and 5 identified as other. The majority (*n* = 114) identified as ODP, 44 identified as nurses and 6 identified as other (textual descriptions included Advanced Practitioner, Advanced Surgical Practitioner or Medical Practitioner with an ODP background).

**Table 2:** Sociodemographic characteristics of the sample

Characteristic		Number or mean (% or *SD*) / *N* = 164
Age	Years	42 (*SD* 11.66)
Gender	Male	37 (22.6)
	Female	112 (68.3)
	Other	5 (3.0)
Profession	Operating Department Practitioner	114 (69.5)
	Nurse	44 (26.8)
	Other	6 (3.7)

Education and professional levels of education currently held by participants provided a wide range of responses indicative of both the ODPs’ and TNs’ historical changes to professionally approved education and training pathways leading to registration (Table 3).

**Table 3:** Education and registration

Characteristics	Number of respondents	Number (%)
Diploma in Higher Education in ODP	49	29.9
City and Guilds 752 Operating Department Assistant	13	7.9
NVQ Level III in Operating Department Practice	17	10.4
BSc in Operating Department Practice	4	2.4
BSc (Hons) in Operating Department Practice	33	20.1
Registered Nurse	19	11.6
Project 2000	3	1.8
BSc Nursing	6	3.7
BSc (Hons) Nursing	12	7.3
MSc Nursing	3	1.8
Missing	5	3.1

Overall, participant experience of research was variable. However, 53 (32.3%) of participants responded that they had undertaken research while in clinical practice working as post-qualified ODP or TN. The majority of participants 110 (67.1%) had not undertaken any research with missing data from 1 (0.6%) participant. It is worth noting that 22 (13.4%) of participants declared that they had an MSc not specific to ODP or Nursing. Examples of these awards were MSc in Surgical Practice, MSc in Advanced Surgical Care and MSc in Advanced Surgical Practice. Notably, 1 (0.6%) participant reported achieving a Doctorate. Data on educational and registration were missing in five (3.1%) responses.

An overview of participant geographical location frequency and percentage can be seen in Table 4.

**Table 4:** Overview of Geographical location of participants

Characteristics	Frequency	Number (%)
Northern Ireland	Antrim: 1	0.6
	Fermanagh: 1	0.6
Northern England	East: 13	7.9
	West: 20	12.2
	Yorkshire and Humber: 20	12.2
Midlands England	East: 14	8.5
	West: 11	6.7
	Anglia: 24	14.6
London	12	7.3
South England	East: 13	7.9
	West: 23	14.0
Scotland	4	2.4
Wales	Southwest: 1	0.6
	Southeast: 1	0.6
None of the above	5	3.0
Missing	1	0.6
Total	164	100.0

Five (3.0%) participants identified with none of the above locations, with locations given, instead, for British Territories, such as Gibraltar. Only one participant (0.6%) did not provide location.

The means and standard deviation for the ATR global measure and subscales are presented in Table 5.

**Table 5:** Mean scores for each scale from whole sample (*N* = 164)

Characteristics	*M*	*SD*
Attitudes to research	4.8257	0.52528
Research usefulness	5.9414	0.95613
Research anxiety	3.9527	0.84639
Positive attitudes to research	5.1765	1.22399
Relevance of research to life	3.8342	0.74743
Difficulty of research	4.1877	0.87776

### Data analyses

The regression model is presented in Table 6. The association between attitudes to research and the predictor variables in the model was moderate (*R* = 0.342). The initial predictor variables were identifying as a woman, holding an MSc or higher degree, age in years and previous experience of involvement with research in practice. Collectively, these variables explained 11.7% of the variance in attitudes to research. The overall model was statistically significant (F4.787, p ⩽ 0.01). Further analysis identified that age and gender did not contribute significantly to the model, but holding an MSc or higher degree and previous experience of doing research did. The standardised coefficients suggested that holding an MSc degree was the most important predictor in the model.

**Table 6:** Multiple regression model top predict variance in attitudes towards research

Model	Unstandardised coefficients B	Standard error	Standardised coefficients Beta	t*	Statistical significance (p)
Constant	4.503	0.209		21.590	<0.001
Age in years	−0.001	0.004	−0.011	−0.133	0.894
Holding an MSc degree or higher	0.297	0.111	0.221	2.670	0.008
Have you undertaken any research while in clinical practice / working as a post-qualified ODP/ Theatre Nurse?	0.232	0.093	0.205	2.506	0.013
Being a woman versus not being a woman	−0.038	0.092	−0.033	−0.417	0.678

Further analyses were conducted to explore the relationship between the different facts of attitudes to research. The enter method regression models provided insufficient evidence to support an association between the same predictor variables and research anxiety, relevance to life and research difficulty as measured by the ATR subscales. However, the models did suggest that there could be some relationship between the predictor variables and research usefulness (p = 0.001) and positive attitude to research (p = 0.001).

Age (0.894) and gender (0.678) did not have a relationship to positive attitudes to research. The overall data for respondents age and gender can be seen in Tables 7 and 8.

**Table 7:** Sociodemographic of respondents in age

Characteristics	N	Minimum	Maximum	Mean	SD
Age	159	18	70	42.08	11.659
Valid N	159				

**Table 8:** Sociodemographic of respondents’ gender identity

Characteristics	Frequency	%	Valid percentage	Cumulative percentage
I identify as a man	37	22.6	22.6	22.6
I identify as a woman	112	68.3	68.3	90.9
Other with option to self-describe	5	3.0	3.0	93.9
Prefer not to say	10	6.1	6.1	100.00

## Discussion

Health Education England (HEE) (2022) suggested that research-active organisations perform better, deliver higher quality of care, have improved patient safety and offer a better patient experience (Comer et al 2022). Research-active organisations are also shown to provide more and greater staff development opportunities, which would support HEE’s AHP Research Strategy and NHS Long-Term Plan (HEE 2022, NHS 2019a) and the more recent Multi-professional Practice-based Research Capabilities Framework (NHS 2024).

This survey on ‘positive attitudes to research’ and the research model used to analyse the emergent data, identify some early evidence suggesting a stronger relationship to positive attitudes to research can be attributed to ODPs’ and TNs’ who are post-qualified / post-graduates. The data indicate that ODPs and TNs who hold an MSc and who have also been involved with or undertaken research while in clinical practice have a more positive attitude to research. In addition, the findings also suggest that there could be some relationship between the predictor variables and research usefulness and a positive attitude towards research.

These findings provide some evidence that it could be beneficial to focus on providing both education on the value and reasons for conducting research, and about research design. Exposure to research during studies and in practice could also help to increase the value placed on research as a part of evidence-based practice. However, more research should be undertaken and data gathered to better inform and understand these early survey findings as participants in this survey were not required to express their reasons for the responses given. For example, ‘belief in usefulness of research’ did not require participants to express this in their own words or what it might look like.

This was a small-scale study which has been useful to indicate useful avenues for further research into improving ODPs’ and TNs’ attitudes to research. While the sample size was small, the number of respondents did exceed the sample estimate due to the support of professional bodies for the survey and was sufficient from a power calculation perspective. Volunteer bias and acquiescence bias may also have arisen with respondents choosing to take part because they already had an interest in research. The data collected also suggested that a minority of participants may have been an ODP or TN by original professional background, but are now a surgeon or Anaesthetists, Surgical Care Practitioner or other AHP, and which would have deemed them ineligible to participate. In addition, it is acknowledged that these findings should be explored further, with a greater number of predictor variables to improve the predictive power of the models and to potentially explore barriers perceived by these professional groups. This initial study has revealed interesting patterns, which highlight the key role that education about research and real-world experience could play in improving staff involvement within research.

## Conclusion

The study was interested in understanding the current attitudes of ODPs and TNs towards research. Analysis of the data suggests that participants holding an MSc degree and having previous experience of research is associated with a more positive attitude to research. When the specific attitudes to research were explored individually, there was also some evidence that the overall positive attitude to research was more likely to be related to a belief in the usefulness of research and prior exposure.

## Recommendations:

The authors suggest that the emergent information from this survey could be used to inform discussion and decision-making in areas such as the following:

- Embracing a robust approach to research and its value within the undergraduate and postgraduate education of ODPs and TNs.
- Educationally, the focus should be on increasing the actual value of research and its usefulness for ODPs and TNs rather than focusing on reducing anxiety of research and its relevance to their personal life.
- Developing career aspirations, opportunities and engagement of ODPs specifically alongside other AHPs and Nursing, in line with current NIHR projects and investment into increasing research activity within AHP careers (NIHR 2023a).
- Consideration of how research is incorporated into ODPs’ and TNs’ clinical and non-clinical job planning activity (NHS 2019b).
- Other possible applications could include informing discussion and decision-making in areas such as:
- Clinical Research: Evidence-Based Practice / patient care and service development
- Local / national / international workforce institutions and professional body strategic thinking (pre- and post-qualification)
- Stronger academic / research and clinical career pathways
- Research aspiration (individual / departmental / institutional /professional)
- Shared clinical and academic development pathways
